# Global warming and malaria: knowing the horse before hitching the cart

**DOI:** 10.1186/1475-2875-7-S1-S3

**Published:** 2008-12-11

**Authors:** Paul Reiter

**Affiliations:** 1 Insects and Infectious Disease Unit, Institut Pasteur, 25-28 rue du Dr Roux, 75724 Paris, France

## Abstract

Speculations on the potential impact of climate change on human health frequently focus on malaria. Predictions are common that in the coming decades, tens – even hundreds – of millions more cases will occur in regions where the disease is already present, and that transmission will extend to higher latitudes and altitudes. Such predictions, sometimes supported by simple models, are persuasive because they are intuitive, but they sidestep factors that are key to the transmission and epidemiology of the disease: the ecology and behaviour of both humans and vectors, and the immunity of the human population. A holistic view of the natural history of the disease, in the context of these factors and in the precise setting where it is transmitted, is the only valid starting point for assessing the likely significance of future changes in climate.

## 

*Malaria is ... a thousand different diseases and epidemiological puzzles. Like chess, it is played with a few pieces, but is capable of an infinite variety of situations*.

*Malaria in Europe, An Ecological Study *[1]

Man-made climate change has become a defining moral and political issue of our age. Speculations on its potential impact often focus on infectious diseases, and on malaria in particular. Predictions are common that in the coming decades, tens – even hundreds – of millions more cases will occur in regions where the disease is already present, and that the vectors and the pathogens will move to higher latitudes and altitudes.

Numerous review publications [2-34] and substantial media attention have had a major impact on public perceptions of the issue. In most cases, these publications make brief mention of where malaria occurs and how it is transmitted, followed by a succession of statements on the action of temperature, rainfall and other climate variables on specific components of the transmission cycle. These statements – often valid in themselves – are used to justify disquieting predictions that are persuasive because they are intuitive. Some are based on mathematical models that select a climate variable (usually temperature), propose a direct interaction with a transmission parameter (e.g. multiplication of the parasite, survival of the vector), and inevitably arrive at the same conclusions. Many focus on the vulnerability of people in poorer countries and place the blame squarely on the activities of the industrial nations. A deplorable trend in the scientific press is the inclusion of a political message, much as in the popular media.

The great majority of these publications sidestep four factors that are key to the transmission and epidemiology of the disease: the ecology and behaviour of humans, and the ecology and behaviour of the vectors. There is rarely any mention of a fifth, the immunity of the host and of the host population. These factors interact with each other, and are themselves made up of an intricate network of highly variable parameters. The true dynamics of transmission can only be assessed by taking this daunting complexity into account. Moreover, the key climate variables – temperature, rainfall and humidity – cannot be viewed independently; the effects of temperature are modified by humidity, the daily range of each may be more significant than the daily mean, brief periods of atypical heat or cold can be more significant than long-term averages, heavy storms can have a different impact than light prolonged rainfall, one year's events may have a significant impact on subsequent years, and so on.

In summary, a holistic view of the complex natural history of malaria in the precise setting where it is transmitted is mandatory for any speculation on the role of climate variables; the significance of these variables, and their putative role in future climates, can only be assessed in the perspective of this complexity.

## Mathematical models

The mathematical modelling of malaria began with Ronald Ross, soon after he had demonstrated the transmission cycle of the parasite. Fifty years later, Macdonald modified his approach to build a more comprehensive model that proved useful in developing strategies for control. Both men were aware that their approach was limited; they recognized that factors such as immunity, behaviour and seasonality were not included, mainly because they are hard to quantify. Nevertheless, many of the claims that are made in the climate change debate blithely present Macdonald's simple deterministic model, list its component variables, point out that the extrinsic incubation period is sensitive to temperature and thereby reason that higher temperatures will mean higher rates of transmission. To the layman, this naïve approach may be persuasive because it is intuitive, but Ronald Ross and Macdonald would certainly not have approved.

The advent of low-cost computers has propelled mathematical modelling into a major role in the description of complex systems, including ecology, epidemiology and public health [35]. It is now relatively simple to run stochastic models, built of a selected set of variables, with interactions driven by sets of differential equations. Complex systems imply the need for a large number of variables and operators, but as these numbers increase, so does the variance – and the uncertainty – of the models. In a sense, therefore, they remain an extension of the intuitive approach because the selection of variables, the assumptions of the frequency distributions that are involved, the mathematical descriptors of the operators, and the constraints on both are made by the modeller. This is not to say that such models are inappropriate. On the contrary, they have become a central component of infectious disease epidemiology, mathematical representations that can be used to explore the dynamics of transmission and the fit of such models to field data. Nevertheless, an inescapable constraint in the case of vector-borne diseases is that the natural history and ecology, of both vector and host, are so complex that it is facile to predict future prevalence and incidence on the basis of temperature.

## Common misconceptions

There is a widespread misconception that mosquito-borne diseases require tropical temperatures, or at least the temperatures of the warmer temperate regions. A glance at a map of global isotherms reveals that summer temperatures in many temperate regions are at least as high as in the warmest seasons of many regions in the tropics. The crucial difference is that the tropics do not have cold winters. Moreover, if tropical mosquito-borne pathogens are introduced to temperate regions in the right season, they can be transmitted, if suitable vectors are present [34].

There is also a misconception that mosquitoes die in winter, and that more die in colder winters, but it is obvious that mosquitoes – and indeed all life-forms that are native to temperate regions – have evolved strategies to survive low temperatures. In the tropics, comparable adaptations are necessary for survival during unfavorable dry periods that may last for several years. In both cases, such adaptations merely impose seasonality on transmission. In southern Europe, for example, *Plasmodium falciparum *(the most pathogenic species of malarial pathogen) was once transmitted from July to September [36]. In Mali, where the disease is still endemic, it is limited to the same three months, the wet season [37].

The physical environment is an important modifier of local climate. *Anopheles arabiensis*, an important vector of malaria in Africa, can survive in the Sudan when outdoor temperatures are above 55°C by hiding in the thatch of buildings in the daytime, feeding after mid-night, and ovipositing at dawn or dusk [38]. In Lapland, in the past, anopheline species survived the winter in houses and stables, feeding occasionally, and transmitting malaria when outdoor temperatures were below -40°C [39].

These examples underline the limited value of meteorological variables as a guide to the behaviour and geographic range of vector species, and of the pathogens they transmit.

## Malaria in temperate regions

Few people are aware that it is less than forty years since the final eradication of malaria in Europe and the United States. Indeed, the disease was common in the period from the 16^th ^to 18^th ^centuries that climatologists term the Little Ice Age [33], and data from burial records around the Thames estuary reveal that mortality in "marsh parishes" of England was comparable to that in areas of transmission in sub-Saharan Africa today [40,41].

**Figure 1:**
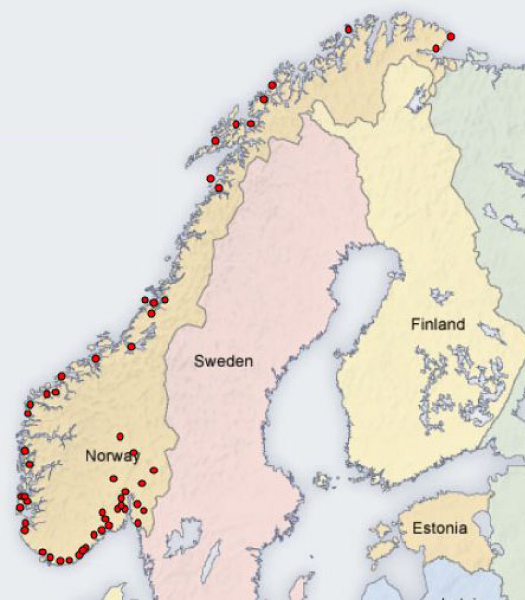
Malaria in Norway, 1860–1920 [57]. Unpublished map by Lena Hulden and Larry Hulden (with permission of the authors).

Until the mid-19^th ^century, the northern limit of transmission was roughly defined by the present 15°C July isotherm. Denmark and parts of Sweden suffered devastating epidemics until the 1860s. Incidence diminished thereafter and the disease had essentially disappeared around the turn of the 20^th ^Century. The same was true in Finland, except for a brief recrudescence in 1941, during the Russo-Finnish war. Figure 1 shows the distribution of malaria cases in Norway between 1860 and 1920. In England, there was a gradual decrease in transmission until the 1880s, after which it dropped precipitously and became relatively rare, except in a short period following World War I. In Germany, transmission also diminished rapidly. After World War I it was mainly confined to a few marshy localities [36].

This regional decline is attributed to a combination of factors:

### Ecological change

Improved drainage, reclamation of swampy land for cultivation and the adoption of new farming methods all served to eliminate mosquito habitat (there is an old Italian saying: "malaria flees before the plough").

### New farm crops

New root crops such as turnips and mangel-wurzels were adopted as winter fodder. These enabled farmers to maintain larger numbers of animals, particularly cattle, throughout the year. European vectors of malaria readily bite cattle, so the increasing size of herds diverted mosquitoes from feeding on humans. Human malaria parasites cannot infect cattle, so this diversion reduced the number of infected mosquitoes and infective bites.

### New rearing practices

Selective breeding of cattle, and new introductions (e.g. the Chinese domestic pig), in combination with the new fodder crops, enabled farmers to keep large populations of stock in farm buildings rather than in open fields and woodland. These buildings provided attractive sites for adult mosquitoes to rest and feed, diverting them from human habitation.

### Urbanization and mechanization

Rural populations declined as industrialization drew people to urban areas. The increased ratio of cattle to people further reduced the attack rates of mosquitoes on humans.

### Human living conditions

New building materials and improvements in construction methods made houses more mosquito-proof, especially in winter, another factor that reduced contact with the vector.

### Medical care

Greater access to medical care, and wider use of quinine (in part due to a major reduction in price) reduced the survival rate of the malaria parasite in its human host, thus limiting the infection rate of mosquitoes.

These factors are a classic illustration of the role of ecology and behaviour in the prevalence and incidence of a mosquito-borne disease. Moreover, the decline cannot be attributed to climate change, for it occurred at the start of the current warming phase. Nor can it be attributed, as is often stated, to deliberate mosquito control, for it came before recognition of the role of the vector.

In contrast, in countries that lagged in these changes, malaria did not decline "spontaneously" [36]. In the Soviet-block countries, for example, from Poland to eastern Siberia, major epidemics occurred throughout the 19^th ^century and the disease remained one of the principal public health problems for the entire first half of the 20^th ^century. Indeed, in the 1920s, in the wake of massive social and economic disruption, a pandemic swept through the entire Soviet Union. Official figures for 1923–25 listed 16.5 million cases, of which not less than 600,000 were fatal [36]. Tens of thousands of infections, many caused by *P. falciparum*, occurred as far north as the Arctic seaport of Arkhangelsk (61° 30^'^N).

The advent of DDT revolutionized malaria control. Cheap, safe, effective applications of the chemical could be targeted at the site where most infections occur – in the home. Initial efforts in Italy, Cyprus and Greece were so successful that a decision was made to eradicate the disease from all of Europe. The entire continent was finally declared free of endemic malaria in 1975. One of the last countries affected was Holland.

Several efficient malaria vectors are indigenous to North America, and are common in many areas. The pathogen probably arrived in the hemisphere in infected European colonists and African slaves. The history of its decline is similar to that of Europe. In the 1880s, it was widespread in nearly all states east of the Rocky Mountains, from the semitropical Gulf Coast states to the northern border and into Canada. It was also present west of the Rocky Mountains, particularly in areas where rainfall is abundant. As living conditions improved, and anti-malarial drugs became more widely available, the incidence of the disease declined. During World War II, the US Military created an Office of Malaria Control in War Areas, responsible for military bases throughout the country. In a symposium published in 1941 [42], the disease was described as "of moderate endemic intensity in the sixteen south-eastern states...with scattered epidemics at intervals of approximately seven years." In 1946 the United States Congress established a new agency, the Communicable Disease Center. This was the forerunner of the U.S. Centers for Disease Control and Prevention (CDC), and its principal mission was to eradicate malaria from the entire country. Its headquarters were in Atlanta, Georgia, because the southern states were the main region still affected by the disease. When operations began, foci of transmission were already hard to find, but the disease was not totally eliminated until the late 1950s. Today, as in Europe, transmission cycles have been disrupted and the pathogens are absent.

Malaria vectors are, of course, still widespread in Europe and North America, and there are occasional cases of autochthonous transmission when an infected traveler infects local mosquitoes. Given the efficacy of anti-malarial therapy, however, and the increasing sophistication of disease surveillance, it is safe to say that there is no chance of significant transmission arising from such cases, and certainly no chance of the disease becoming endemic, at least under current economic circumstances.

## Malaria in the tropics

Ninety percent of the estimated 300–500 million cases of malaria worldwide occur each year in sub-Saharan Africa. Statements on climate change and human health often focus on this region, with predictions that, by the mid-21^st ^century, tens of millions more cases will occur there as a direct result of increasing temperatures (see for example [2]).

A critical aspect of malaria epidemiology is the concept of *stability*. In much of sub-Saharan Africa, parts of northern India, Indonesia, South America and elsewhere, transmission is termed *stable *because it is fairly constant from year to year; the disease is endemic, but epidemics are uncommon. In other regions, including parts of India, Southeast Asia, Central and South America, the disease is also endemic but is termed *unstable *because transmission can vary greatly from year to year, and the potential for epidemics is high. These terms are, of course, a simplification; there is a wide range of degrees of stability, depending on complex factors in local circumstances.

### Stable endemic malaria

In regions where the principal malaria vectors are anthropophilic (i.e. prefer to feed on humans) and have a high survival rate, transmission is usually stable. The disease is hard to control because transmission is efficient and transmission rates are so high that most people experience many infective bites per year. Severe illness and mortality occurs mainly among "new arrivals," i.e. children and non-immune immigrants. Older inhabitants have survived multiple infections and maintain a degree of immunity by repeated re-infection. They can have bouts of illness that may be life-threatening, but are usually relatively mild.

### Unstable endemic malaria

This generally occurs in regions where the anophelines are zoophilic (i.e. bite animals as well as humans), or their survival rates are low, or where both apply. Transmission can vary greatly from year to year, with epidemics separated by many years of relatively low activity during which the overall immunity of the population declines. The factors that precipitate such epidemics are often difficult to identify. The disease may appear suddenly, for no apparent reason, only to disappear again without obvious cause. In general, transmission is relatively inefficient and, therefore, requires high mosquito populations. In theory this implies that the disease is easier to control. In practice, it can be catastrophic, not least because the attack rate is not restricted to "new arrivals".

**Figure 2:**
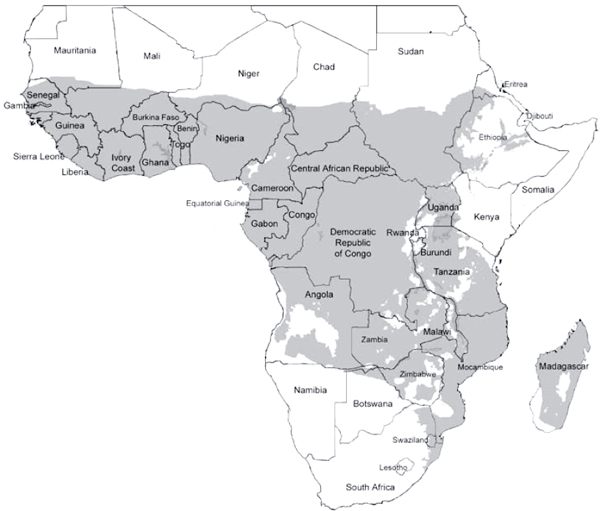
Current suitability of local climatic conditions for stable transmission of malaria in sub-Saharan Africa, i.e. regions where significant transmission is likely to occur every year. Epidemic transmission is mainly limited to narrow bands to the north and south of the shaded area. Adapted from a map based on available long-term climate data published by the MARA/ARMA project [58].

The vast majority of people in sub-Saharan Africa live in regions of stable transmission. In other words, throughout their lives, people living in the shaded areas of the map (Figure 2) are regularly exposed to multiple bites from infective mosquitoes, as many as 300 infective bites per year in some regions. Under such circumstances, just as it is impossible to pour more water into a glass that is already full, it is illogical to suggest that increased temperatures will result in an increased incidence of infections. On the other hand, changes in rainfall patterns could alter stability and distribution.

Thus, as in temperate regions, the behaviour and ecology of vector and host are the dominant factors in transmission. In many cases, these are attributable to explosive population growth and poverty.

### Birth rate

The world's population has grown from 2.5 billion in 1950 to 6.7 billion at the time of writing (September 2008). In sub-Saharan Africa, there are now nearly five times as many people (ca. 750 million) as there were in 1955. In some countries, more than half the population is under 15 years of age. High birth rates often give rise to larger communities with higher densities of people, which can lead to a higher attack rate; clinical studies in some parts of Africa quote 998 infections per 1,000 infants.

### Forest clearance

Many malaria vectors breed in open, sunlit pools. Forest clearance provides abundant new habitat for these species, a classic cause of the emergence of malaria problems [43].

### Agriculture

Irrigation creates an ideal habitat for mass-production of mosquitoes, as can construction of dams for hydroelectric power. Rice cultivation provides an environment for many of the most efficient malaria vectors. Conversely, the cultivation of ground depressions can suppress such vectors and thereby reduce transmission [].

### Movement of people

Infected people in pursuit of work can introduce malaria to areas where it is rare. Non-immune people are at high risk if they move to areas of transmission. Extensive road building and modern transportation have greatly exacerbated this factor.

### Urbanization

Water storage and inadequate water disposal can provide habitat for mosquitoes, particularly in rapidly expanding urban areas. The absence of cattle can promote stable transmission by forcing zoophilic species to feed on people. Moreover, many tropical cities are surrounded by densely-populated, satellite settlements that are essentially rural in nature.

### Insecticide resistance

*Physiological resistance *to insecticides is common in many regions. *Behavioural resistance *can also be a major problem: species that prefer to feed and rest indoors (*endophilic*) can switch to outdoor (*exophilic*) activity in response to treatment of indoor surfaces with insecticides.

### Drug resistance

In many parts of the world, the malaria parasite has evolved resistance to commonly used anti-malarial drugs. Substitutes are available, but are much more expensive.

### Degradation of the health infrastructure

Lack of funding, institutional difficulties, rapid urbanization and other problems associated with rapid development have eroded the public health sector of many countries. In addition, the AIDS pandemic has overwhelmed the ability of authorities to deal with other diseases.

### War and civil strife

In times of conflict, mass movements of people, e.g. soldiers and refugees, often promote malaria transmission. The breakdown of public health services, damage to water distribution and drainage systems, and the destruction of homes often exacerbate the situation. High concentrations of people in camps for displaced persons can also be disastrous.

## Highland malaria in the tropics

A topic that is repeatedly cited in the climate change debate, both in the scientific and the popular press, is that warmer temperatures will drive malaria transmission to higher altitudes in the tropical highlands, particularly East Africa. Indeed, environmental activists frequently state that this is already happening.

It is certainly true that the incidence of malaria has increased in some highland areas, and it is perfectly acceptable to cite temperature as a limiting factor in transmission; vectors such as *Anopheles gambiae *have been reported at altitudes up to 3,000 m above sea level, but endemic malaria disappears above 1,800–2,000 m. What is rarely mentioned is that less than two percent of the African continent (including North Africa) is above 2,000 m, and that much of this is so arid that it offers little opportunity for cultivation. Moreover, the history of malaria in highland areas is a compelling example of the dominant role of human behaviour and human ecology, not climate, as the driving factors in the dynamics of transmission.

Two examples illustrate this point: the Kenya Highlands and the New Guinea Highlands.

## Kenya Highlands

Nairobi, capital of Kenya, was founded in 1899 during the construction of a railway from Mombassa, on the coast, to Lake Victoria. The site was chosen because it was on the last stretch of level ground before the steep descent into the Rift Valley. It was a swampy area, and had always been known as an unhealthy locality "swarming with mosquitoes" [45]. Indeed, in 1904, when the town had already grown substantially, a committee of doctors petitioned the colonial administration that the entire municipality be relocated because it was a spawning ground for disease. At that time it marked the upper limits (1,068 m) of malaria transmission in the region, but the disease began to appear at higher altitudes after the clearance of forests for the development of tea estates and the importation of infected labourers [46]. The first sizeable epidemic, shortly after World War I, was attributed to the return of local soldiers from Tanzania. A major epidemic in 1926 led to recognition that economic development was a key factor in the proliferation of mosquito breeding sites, and hence the source of the increasingly serious problem:

*That there have been no notable general alterations in the domestic environment of the natives of these reserves during recent years is true, but on the other hand it is to be remembered that in every direction roads, and to a lesser extent, railways, have been carried into and through these areas, and always where there are roads, artificial and un-drained excavations are to be found *[47] quoted by Snow *et al *[48]

In the following year, the colonial administration and the Municipal Corporation of Nairobi agreed to spend £40,000 (the equivalent of nearly £800,000 (US$ 1,400,000) in current currency) for eradication of anopheline breeding sites in the Nairobi area. Nevertheless, there were six major epidemics in the city between the two World Wars, with serious rates of transmission extending to the Londiani district (2,250–2,490 m) and even at a farm near Mount Timboroa, at 2,490–2,550 m [49].

The fundamental cause of the upward advance of the disease was widespread deforestation and development as the areas were opened up for large farming ventures. As in the quote above, the construction of roads and railways created innumerable flooded "borrow pits" (depressions left by excavation for materials) and also contributed to the dispersal of the mosquito. The introduction of the ox-wagon caused a proliferation of rough cart roads; water in the wheel ruts provided a prolific breeding site for vectors. Mill-dams on rivers interfered with natural drainage [49].

These and many other factors were components of a drastic ecological change, and it was this change that brought transmission to the Highlands. The disease continued to be a serious public health problem until the 1950s, when the administration organized an extensive control programme, mainly based on DDT, after which the area was essentially malaria-free until the 1970s.

The tea-growing estates in the Kericho district (1,780–2,225 m) have an extensive medical service for employees and their dependents that was initiated in 1925. Health care at the central hospital of Unilever Kenya Ltd. is extended to some 100,000 inhabitants of the region. However, there is no attempt at mosquito control, and malaria has re-emerged as a serious problem. Epidemics occurred almost every year from 1990 to 1997, with a mean annual attack rate of around 50 percent [50]. Peak transmission was from May to July, after the principal rainy season and before mean monthly temperatures drop below 18°C. A questionnaire survey (June 1997) indicated that only 8 percent of patients had travelled to areas with known malaria transmission in the previous 30 days.

The main factor in this recrudescence may be increased resistance to anti-malarial drugs, as well as the unsupervised use of ineffective medications, but the picture is not entirely clear [51]. Whatever the cause, the history of multiple epidemics in the earlier part of the century, including many at higher altitudes, makes it unnecessary to infer climate change as a contributory factor. Moreover, a set of well-maintained meteorological records shows no significant change in temperature over recent decades [52]. Indeed, in a detailed report to the World Health Organization, a group of malaria specialists based in Nairobi dismissed those who claim a global warming link as "scientific Nostradamuses" [48].

## New Guinea Highlands

In the early 1930s, a human population estimated at 1,000,000, previously unknown to outsiders, was contacted in the mountains of New Guinea. It appeared that these so-called "Stone Age people" were malaria-free, and this was attributed to their unique state of isolation. By contrast, the lowland coastal regions were highly malarious.

At first, the highlanders became a new source of labour for the coastal plantations, but after World War II there was a rapid increase in the number labourers from the lowlands employed by landowners growing *Arabica *coffee and other crops in the mountains. In the late 1940s, government scientists warned that the increasing contact between the regions could bring disaster, for epidemic malaria had already appeared in several highland areas at around 1,500 m [53]. By the mid-1950s several alarming outbreaks lead to the enforcement of a law that required employers of highlanders working on the coast to supply them with anti-malarials, and to ensure that the medications were actually taken. On repatriation, highlanders were held by the government in compulsory quarantine for two weeks and given curative malaria therapy [54,55].

These regulations failed to stop the emergence of the disease and its spread to many isolated valleys. As in Kenya, the increase in prevalence was clearly attributable to a rapid increase of anopheline populations after forest clearance, the influx of people from malarious areas, and the construction of roads, airstrips, plantations, mines, water impoundmentsand other human artifacts. Several vectors were involved, including *Anopheles farauti *and *Anopheles punctulatus *[56]. These species breed in open sunlit pools and are essentially the local equivalent of *An. gambiae *and *Anopheles funestus *in sub-Saharan Africa.

Subsequent studies suggest that the parasite may have arrived in the highlands as early as the 1940s but did not become evident until forest clearance and development were more widespread. Whatever the chronology, the history of the disease is clearly attributable to the introduction of the parasite to non-immune populations and the proliferation of its vectors as a result of large-scale ecological change.

## Conclusion

Simplistic reasoning on the future prevalence of malaria is ill-founded; malaria is not limited by climate in most temperate regions, nor in the tropics, and in nearly all cases, "new" malaria at high altitudes is well below the maximum altitudinal limits for transmission. Future changes in climate may alter the prevalence and incidence of the disease, but obsessive emphasis on "global warming" as a dominant parameter is indefensible; the principal determinants are linked to ecological and societal change, politics and economics. There is a critical need for cheap, effective control campaigns, as were implemented during the DDT era. A creative and organized search for new strategies, perhaps based on new technologies, is urgently required, irrespective of future climate change.

## Competing interests

The authors declare that they have no competing interests.
